# First person – Heta Mattila

**DOI:** 10.1242/bio.061610

**Published:** 2024-07-09

## Abstract

First Person is a series of interviews with the first authors of a selection of papers published in Biology Open, helping researchers promote themselves alongside their papers. Heta Mattila is first author on ‘ Flavonols do not affect aphid load in green or senescing birch leaves but coincide with a decrease in Photosystem II functionality’, published in BiO. Heta conducted the research described in this article while a postdoctoral fellow in Esa Tyystjärvi's lab at University of Turku, Turun Yliopisto, Finland. She is now a postdoctoral fellow in the lab of Sónia Cruz at University of Aveiro, Portugal, investigating the effects of high light stress on different photosynthetic organisms.


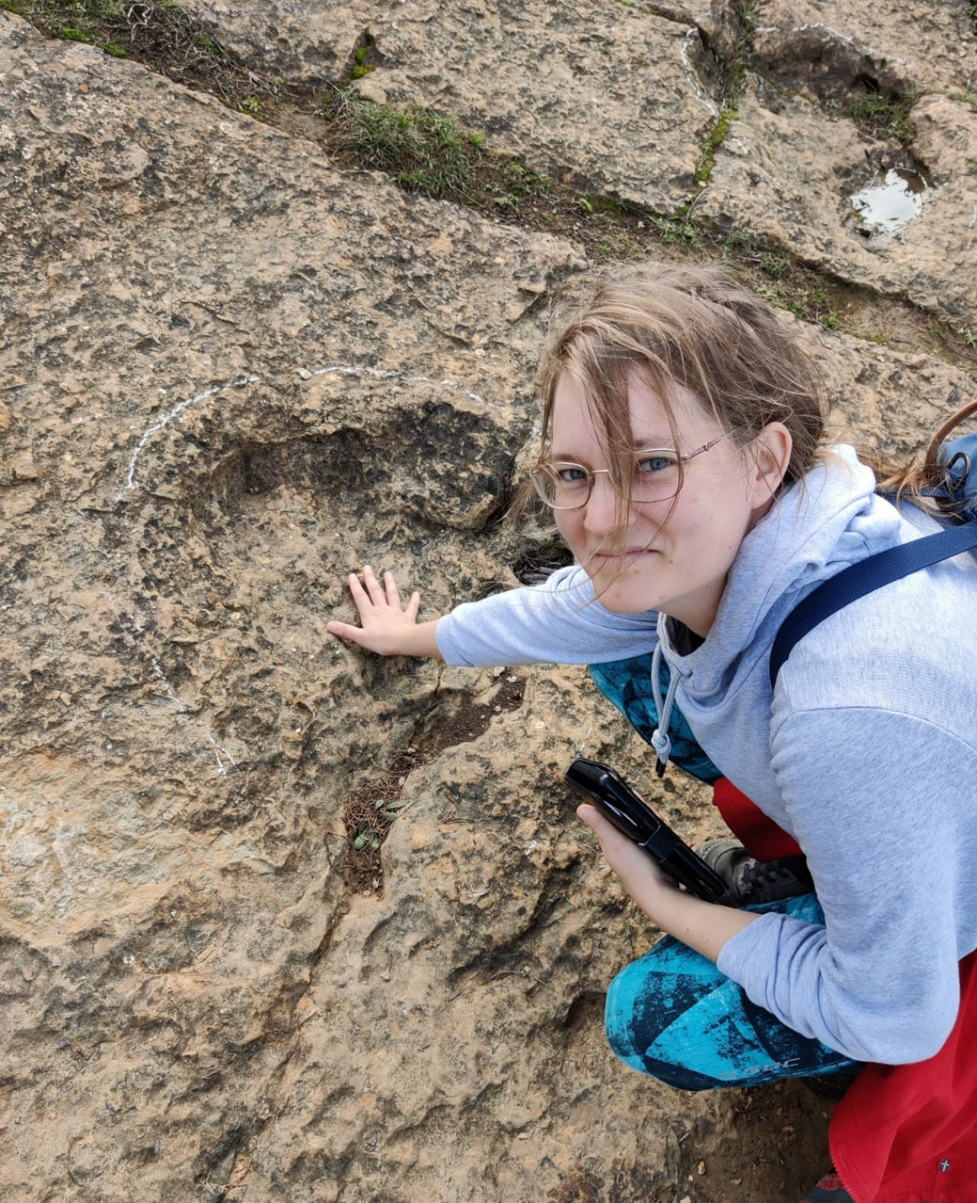


**Heta Mattila**

**Describe your scientific journey and your current research focus**

I did my masters’ and doctoral theses at the University of Turku (Finland), at the Molecular Plant Biology unit, focusing on how light damages plants and how plants respond to different lights. Almost the whole time, I also studied tree senescence, as a side project. After defending my doctoral thesis, I continued a bit in Turku, and then moved to Aveiro (Portugal), where I currently am doing my second postdoc. I still work on light stress, but now in addition to plants, I am also studying macroalgae.

**How would you explain the main finding of your paper?**

Through years, there have been many scientific debates about why some plants turn red during autumn. These red pigments are actually synthetized by the dying leaves, and the same leaves can also make pigments that are invisible to the human eye. Birch leaves turn yellow, not red, in the autumn, but simultaneously they produce flavonol pigments. These pigments are invisible to humans but may not be invisible to insects. In respect to autumn, these pigments are little studied. We wanted to know if birch would use them as aphid (an insect harmful to many trees) deterrent. However, we found no evidence for that. Instead, these pigments accumulate more in leaves that show certain stress symptoms.

Through years, there have been many scientific debates about why some plants turn red during autumn.

**Figure:**
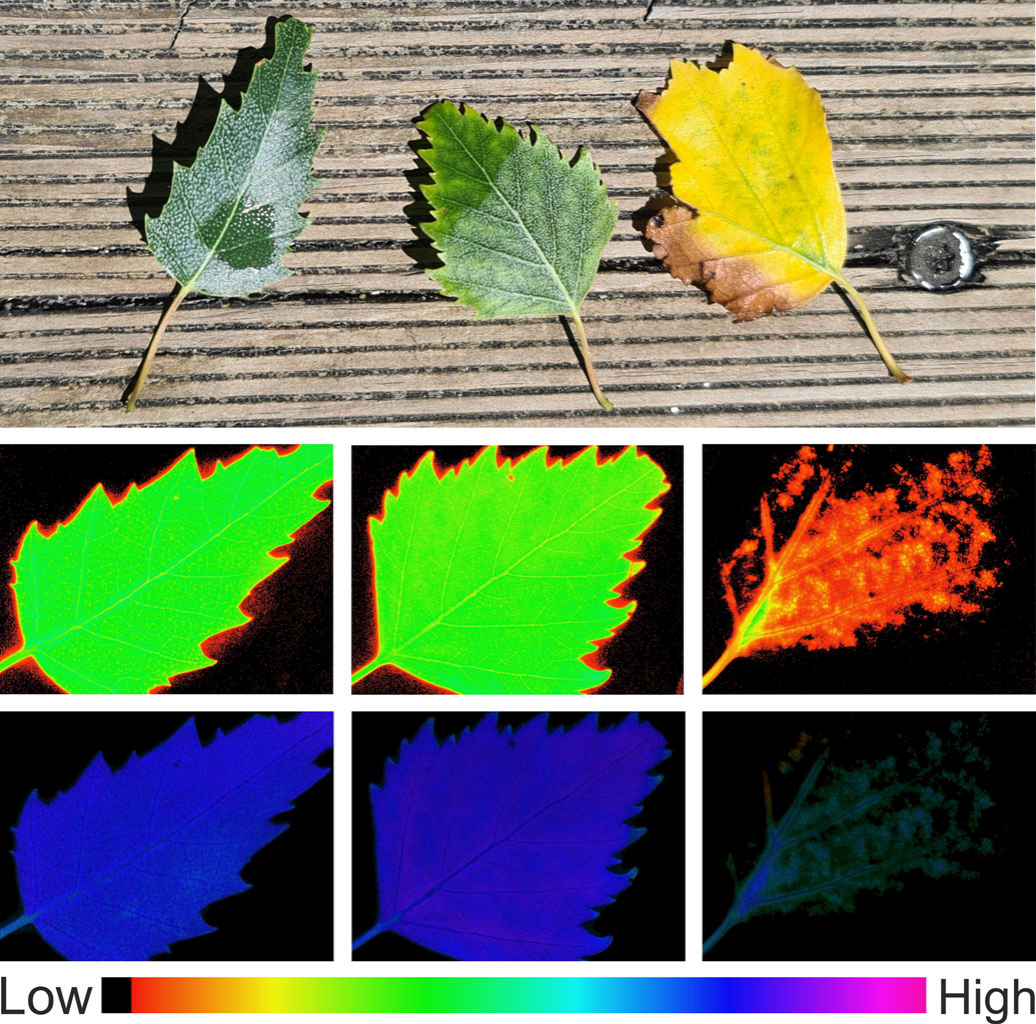
**Photosynthesis probed by chlorophyll fluorescence.** At the end of their lives, senescing (yellow) leaves have very little chlorophyll left, reflected in the low yield of the fluorescence signal (middle row), but those parts that do have some chlorophyll left still exhibit high photosynthetic activity (bottom row). Fluorescence images were taken with the Imaging-PAM fluorometer (Walz, Germany).

**Which part of this research project was the most rewarding?**

I enjoyed the whole journey. When I was a bachelors student, I think, my supervisor was wondering if, in the autumn, trees degrade chlorophyll during the night-time (which would be safer); we tried to measure this but did not succeed. Some years later, I thought that we should try again (with a different methodology). Well, we found out that, actually, chlorophyll is preferentially degraded during the day (maybe to ensure enough energy)! But we also noticed that in birch, at the same time when chlorophyll is degraded, flavonols increase. But why? A few ideas and papers later, here we are with the present work. This is how I like to do research; you make an observation, you start to pursue it, and finally, maybe after many years, you solve the mystery! Or maybe don't… This may not be the most straight-forward way but, for me, it is the most enjoyable way. I also believe that if we don't do things “just” out of curiosity, many great discoveries never will be made. Indeed, some of the best papers I have been involved in have been born like this; not because somebody is telling you what to do, but because you find something odd and you want to study it further.

This is how I like to do research; you make an observation, you start to pursue it, and finally, maybe after many years, you solve the mystery!

**What do you enjoy most about being an early-career researcher?**

The freedom. I have my own grant, so I am also my own boss. Of course, there are down-sides to that too, but I like the fact that I am free to pursue those research questions that I think are the most interesting ones. I can also pick up new research directions, or weird side-projects, if I feel that they are more important than the ones I originally was planning to follow.
